# Comprehensive CircRNA Profiling and Selection of Key CircRNAs Reveal the Potential Regulatory Roles of CircRNAs throughout Ovarian Development and Maturation in *Cynoglossus semilaevis*

**DOI:** 10.3390/biology10090830

**Published:** 2021-08-26

**Authors:** Jing Li, Bao Shi, Chongnv Wang, Changwei Shao, Xuezhou Liu, Daiqiang Zhang

**Affiliations:** 1 Key Laboratory of Sustainable Development of Marine Fisheries, Ministry of Agriculture and Rural Affairs, Yellow Sea Fisheries Research Institute, Chinese Academy of Fishery Sciences, Qingdao 266071, China; jleeing@163.com (J.L.); wangchongnv@ioz.ac.cn (C.W.); shaocw@ysfri.ac.cn (C.S.); Liuxz@ysfri.ac.cn (X.L.); zhang1995882021@163.com (D.Z.); 2 Laboratory for Marine Fisheries and Food Production Processes, Pilot National Laboratory for Marine Science and Technology (Qingdao), Qingdao 266237, China; 3 College of Fisheries, Tianjin Agricultural University, Tianjin 300384, China

**Keywords:** RNA-seq, CircRNA, *Cynoglossus semilaevis*, ovarian maturation, reproduction

## Abstract

**Simple Summary:**

CircRNAs: as molecules involved in gene regulation, have become a new research hotspot in the non-coding RNA field. CircRNAs show tissue- or developmental stage-specific patterns of expression and can influence the expression levels of their parental genes. Recent studies have documented the potential biological roles of circRNAs in the growth, development, reproduction and health of humans and animals. Tongue sole (*Cynoglossus semilaevis*) is a marine flatfish that is an economically important farmed species in China. The commercial aquaculture of tongue sole has developed in the last few years because wild resources have decreased. Reproduction is regulated by brain-pituitary-gonad-liver axis which limits the development of artificial tongue sole culture. However, the roles of circRNAs in the ovarian development and maturation of tongue sole has never been reported. The identification of the potential functions of circRNAs provides a foundation for understanding the genetic mechanisms that regulate oocyte growth and maturation, which will allow the efficiency of tongue sole reproduction to be improved. Moreover, our findings extend the knowledge about a new type of endogenous RNA involved in regulating the ovarian development and maturation of tongue sole.

**Abstract:**

CircRNAs are novel endogenous non-coding small RNAs involved in the regulation of multiple biological processes. However, little is known regarding circRNAs in ovarian development and maturation of fish. Our study, for the first time, provides the genome-wide overview of the types and relative abundances of circRNAs in tongue sole tissues during three ovarian developmental stages. We detected 6790 circRNAs in the brain, 5712 in the pituitary gland, 4937 in the ovary and 4160 in the liver. Some circRNAs exhibit tissue-specific expression, and qRT-PCR largely confirmed 6 differentially expressed (DE) circRNAs. Gene Ontology and KEGG pathway analyses of DE mRNAs were performed. Some DE circRNA parental genes were closely associated with biological processes in key signalling pathways and may play essential roles in ovarian development and maturation. We found that the selected circRNAs were involved in 10 pathways. RNase R digestion experiment and Sanger sequencing verified that the circRNA had a ring structure and was RNase R resistant. qRT-PCR results largely confirmed differential circRNA expression patterns from the RNA-seq data. These findings indicate that circRNAs are widespread in terms of present in production-related tissues of tongue sole with potentially important regulatory roles in ovarian development and maturation.

## 1. Introduction

Circular RNAs (circRNAs) are a new class of non-coding RNAs (ncRNAs) with a covalently closed continuous loop structure formed by back-spliced circularization that lack 3′ polyadenylated tails and 5′ polarities [1]. CircRNA production mechanisms demonstrate that exon circularization is dependent on flanking intronic complementary sequences. Moreover, the efficiency of exon circularization is regulated by competition between RNA pairing across flanking introns or within an individual intron [2]. Compared with linear RNAs, circRNAs have the remarkable characteristic of non-canonical splicing, increased stability and resistance to RNase R [1,3,4]. To date, an increasing number of circRNAs have been identified in various tissues and species by high-throughput deep sequencing. Moreover, increasing evidence has clearly shown that circRNAs exhibit strong biological functions in transcriptional and post-transcriptional regulation, and they may be involved in important biological processes [5]. CircRNAs can act as competing endogenous RNAs (ceRNAs), also known as miRNA sponges, to regulate gene expression and circRNAs can also regulate the function of RNA-binding proteins [3,6]. Circ-CSPP1 acts as a miR-1236-3p sponge and impairs the inhibitory effect of miR-1236-3p on ZEB1, which subsequently promotes epithelial-mesenchymal transition and ovarian cancer development [4]. CircEGFR may act as a sponge for miR-125a-3p, thus modulating *Fyn* expression and may play a critical role in ovarian granulosa cells of mice [6]. CircRNA_103827 and circRNA_104816 were predicted to participate in ovarian steroidogenesis and to be ideal biomarkers of ovarian reserve [7,8].

Until recently, an increasing number of studies on circRNAs in fish have been carried out and reported. These studies confirmed the existence of circRNAs in several species, such as zebrafish (*Danio rerio*) [1,9], large yellow croaker (*Larimichthys crocea*) [10], tongue sole (*Cynoglossus semilaevis*) [11], olive flounder (*Paralichthys olivaceus*) [12], grass carp (*Ctenopharyngodon idella*) [13], Nile tilapia (*Oreochromis niloticus*) [14], European Sea Bass (*Dicentrarchus labrax* L.) [15] and medaka (*Oryzias latipes*) [16]. These findings suggest that circRNAs perform certain biological functions in fish. Previous studies have demonstrated that circRNAs are associated with follicular development, ovarian senescence, spermatogenesis and germ cell development, suggesting that circRNAs may function in germ cell regulation [17,18,19].

Tongue sole is a commercially valuable flatfish in Chinese coastal areas. Tongue sole exhibits some degree of reproductive dysfunction under rearing conditions. The major obstacle for large-scale aquaculture is the control of reproduction in captive stocks, especially females, which often show a low oocyte maturation rate [20,21]. Thus, it is important to improve our understanding of the reproductive endocrinology of tongue sole and enhance the reproductive efficiency. However, the relationship between circRNAs and the reproductive regulatory mechanism of tongue sole has not been reported. To our knowledge, this is the first study to elucidate the potential role of circRNAs and their underlying mechanisms during the process of ovary development and maturation in tongue sole, and our data will lay the foundation to characterize the transcriptional regulatory mechanism modulating fish oocyte maturation in the future.

## 2. Materials and Methods

### 2.1. Sample Preparation

According to the histological characteristics described by Shi et al., (2015), the gonads of adult female tongue soles were divided into three developmental stages in this study [20]. The stages of ovarian development of tongue sole were as follows: stage IV, late vitellogenesis; stage V, maturation stage; stage VI, after ovulation. We collected samples of four tissues, namely, the brain, pituitary, liver and ovary, from tongue sole at the three different ovarian stages. The 27 tongue sole for each ovarian stage were collected. Total, 81 tongue sole were collected for RNA sequencing.

The collected tissues were snap-frozen in liquid nitrogen for 10 min and then stored in a −80 °C freezer for subsequent RNA extraction. All procedures involving sample handling and treatment used in this study were conducted in accordance with the relevant guidelines and regulations of the Animal Care and Use Committee of the Chinese Academy of Fishery Sciences (IACUC Issue No. 201933; Approval date: 14 March 2019).

### 2.2. Strand-Specific Library Construction and Sequencing

RNA sequencing was performed in triplicate for four tissues (brain, pituitary, liver and ovary) at ovarian stage IV, V and VI, yielding a total of 36 rRNA-depleted libraries. After total RNA was extracted from brain, pituitary, liver and ovary, ribosomal RNAs (rRNAs) were removed to enrich mRNAs and ncRNAs. Using fragmentation buffer and reverse transcription into cDNA with random primers, the enriched mRNAs and ncRNAs were fragmented into short fragments. Second-strand cDNA was synthesized by DNA polymerase I, RNase H, dNTP (dUTP instead of dTTP) and buffer (Illumina Ribo-Zero Gold (Human/Mouse/Rat) kit (NEB E7490L, New England Biolabs, Ipswich, MA, USA). Next, the cDNA fragments were purified with a QiaQuick PCR extraction kit (Qiagen, Hilden, Germany), end repaired, poly(A) added and ligated to Illumina sequencing adapters. Then, uracil-N-glycosylase (UNG) was used to digest the second-strand cDNA. The digested products were size selected by agarose gel electrophoresis and PCR amplified. Sequencing experiment was conducted by using Illumina HiSeq^TM^ 4000 system (Gene Denovo Biotechnology Co, Guangzhou, China). The RNA-seq data was deposited in NCBI′s Short Read Archive (SRA) under BioProject ID PRJNA698759 for further analysis.

### 2.3. Sequencing Assessment of CircRNA

Reads obtained from the Illumina HiSeq^TM^ 4000 platform included raw reads containing adapter sequences or low-quality reads, which would affect the subsequent analysis. Thus, to obtain high-quality clean reads, the raw reads were further filtered according to the following rules: (1) removing reads containing adapters; (2) removing reads containing more than 10% unknown nucleotides (N); and (3) removing low-quality reads containing more than 50% low-quality (Q-value ≤ 20) bases.

Different species and varying sample quality can affect the efficiency of experimental rRNA removal. Thus, the short read alignment tool Bowtie2 (2.2.8) (https://source forge.net/projects/bowtie-bio/files/bowtie2/2.2.8/ (accessed on 1 March 2016)) was used for mapping reads to the rRNA database. The rRNA-mapped reads were removed, and the remaining reads were further used in alignment and analysis.

The rRNA-free reads of each sample were then mapped to the reference genome of *C. semilaevis* by TopHat2 (version 2.0.3.12) (http://ccb.jhu.edu/software/tophat (accessed on 1 March 2016)). After alignment with the reference genome of *C. semilaevis*, the reads that could be mapped to the genomes were discarded, and the unmapped reads were then collected for circRNA identification.

### 2.4. CircRNA Identification

Next, 20mer sequences from both ends of the unmapped reads were extracted and aligned to the *C. semilaevis* genome to find unique anchor positions within the splice site. Anchor reads that aligned in the reversed orientation (head-to-tail) indicated circRNA splicing and were then subjected to find_circ (version 1.1) to identify circRNAs. The anchor alignments were then extended such that the complete reads aligned and the breakpoints were flanked by GU/AG splice sites. A candidate circRNA was called if it was supported by at least two unique back-spliced reads in at least one sample. The circRNAs were further classified into six types as follows: annot_exon circRNA (the pair of breakpoints was located in the starting point of an exon and the end point of another exon and the circRNA originated from the multiple exons between the breakpoints); antisense circRNA (the circRNA made up of bases on the antisense strand); exon_intron circRNA (the circRNA was circularized with both exons and introns at the same time); one_exon circRNAs (the pair of breakpoints was located in a same exon and the circRNA originated from the nucleotides between the breakpoints); intronic circRNA (the circRNA composed of two or more non-coding RNAs from introns); and intergenic circRNA (the circRNA contained two intronic circRNA fragments flanked by GT-AC splicing signals acting as the splice donor and acceptor of the circular junction while forming an integrated circRNA).

### 2.5. CircRNA Statistics

The identified circRNAs were subjected to statistical analysis of type, chromosome distribution and length distribution.

### 2.6. Functional Enrichment Analysis of Parental Genes

CircRNAs are produced from their parental genes, and analysis of the parental genes can provide valuable insights into the function of circRNAs. First, all parental genes were mapped to Gene Ontology (GO) terms in the GO database (http://www.geneontology.org/ (accessed on 1 March 2016)), gene numbers were calculated for every term, and significantly enriched GO terms in parental genes compared to the genome background were defined by hypergeometric testing. Kyoto Encyclopedia of Genes and Genomes (KEGG; http://www.kegg.jp (accessed on 4 February 2016)) pathway enrichment analysis identified significantly enriched metabolic pathways or signal transduction pathways in parental genes compared with the whole genome background.

### 2.7. Analysis of Differentially Expressed CircRNAs

To identify differentially expressed circRNAs across stages IV, V and VI in the brain, ovary, pituitary and liver of tongue sole, the edgeR package (http://www.rproject.org/ (accessed on 1 March 2016)) was used. We identified circRNAs with a fold change (FC) ≥ 2 and a *p* value < 0.05 in a comparison across stages IV, V and VI in the brain, ovary, pituitary and liver of tongue sole as significant differentially expressed circRNAs.

### 2.8. Database Annotation of CircRNAs

CircRNAs were blasted against circBase (http://www.circbase.org/ (accessed on 15 December 2015)) for annotation. Those that could not be annotated were defined as novel circRNAs.

### 2.9. MiRNA Sponge Analysis and Integrated Analysis of CircRNA-miRNA-mRNA

For circRNAs that have been annotated in circBase, the target relationship with miRNAs was predicted by StarBase (v2.0) (http://starbase.sysu.edu.cn/mrnaCeRNA.php (accessed on 1 March 2016)). For novel circRNAs, three software programmes, Mireap, Miranda (v3.3a) (http://www.microrna.org/microrna/home.do (accessed on 1 March 2016)) and TargetScan (version: 7.0) (http://www.targetscan.org/vert_70/ (accessed on 1 March 2016)), were used to predict targets in the brain, ovary, pituitary, liver of tongue sole at ovarian stages IV, V and VI.

For the prediction of mRNAs interacting with circRNAs and miRNAs, miRTarBase (v6.1) was used to predict mRNAs targeted by miRNA sponges. The resulting circRNA-miRNA-mRNA correlations were visualized by Cytoscape (v3.6.0) (https://cytoscape.org/ (accessed on 4 February 2016)).

### 2.10. RNase R Treatment and Sanger Sequencing

For RNase R treatment, ribosome-depleted RNA was incubated for 30 min at 37 °C with 5 units RNase R per μg RNA (Epicentre Technologies, Madison, WI, USA). Canonical and non-canonical circRNAs were validated by RNase R resistance and sequencing. After RNase R treatment, total RNA from the brain of tongue sole was digested with (R^+^, 30 min at 37 °C) or without (R^−^, water instead) RNase R [22,23].

Then, an aliquot of RNase R-digested RNA was reverse transcribed by an Evo M-MLV RT Kit with gDNA Clean for qPCR (Accurate Biotechnology Co., Ltd, Hunan, China) using random hexamers according to the manufacturer’s protocol, and then reverse-transcription PCR (RT-PCR) amplification was performed with the primers shown in Table S1. The divergent primer can amplify only circRNA molecules and cannot amplify linear RNA. The RT-PCR amplification products were visualized by agarose gels electrophoresis. The amplification product from the each circRNA was obtained by Sanger sequencing at Sangon Biotech (Shanghai, China).

### 2.11. Quantitative Real-Time PCR Analysis of CircRNAs

Total RNA of the brain, ovary, pituitary and liver of tongue sole (n = 3) at staged IV, V and VI was isolated using TRIzol reagent (Accurate Biotechnology Co., Ltd, Hunan, China), reverse transcribed to cDNA using random primers and used as a template for quantitative real-time PCR (qRT-PCR). qRT-PCR detection was performed using the SYBR Green Premix Pro Taq HS qPCR Kit (SYBR Green Premix Pro Taq HS qPCR Kit) in a Mastercycler ep realplex real-time PCR instrument (Eppendorf, Germany). The amplification reaction parameters were as follows: denaturation at 95 °C for 2 min, followed by annealing extension at 95 °C for 15 s and 60 °C for 30 s for a total of 40 cycles. To determine the amplification efficiency and expression levels of circRNAs using *β-actin* gene as the internal control, the comparative delta-delta CT method (2^−ΔΔCt^) method [20] was used, and all reactions were performed in triplicate. Sanger sequencing of qRT-PCR products further verified the circRNA sequence. The primers used are shown in Table S2. 

## 3. Results

### 3.1. Expression Profiles of CircRNAs in the Brain, Ovary, Pituitary and Liver at Different Ovarian Stages

The sequencing of circRNAs from *C. semilaevis* brain, pituitary, liver and ovary tissue was performed at ovarian stage IV, V and VI, yielding a total of 36 rRNA-depleted libraries. On the basis of high-throughput sequencing data of circRNA (Table S3), all the sequencing samples were subjected to filtering low quality sequence to obtain approximately 97% high-quality clean reads, all with Q20 values over 96% and Q30 value above 90%. Overall, the sequencing data of all samples in this study were at over 90% high quality and met the high credibility requirements for subsequent data analysis.

**Figure 1:**
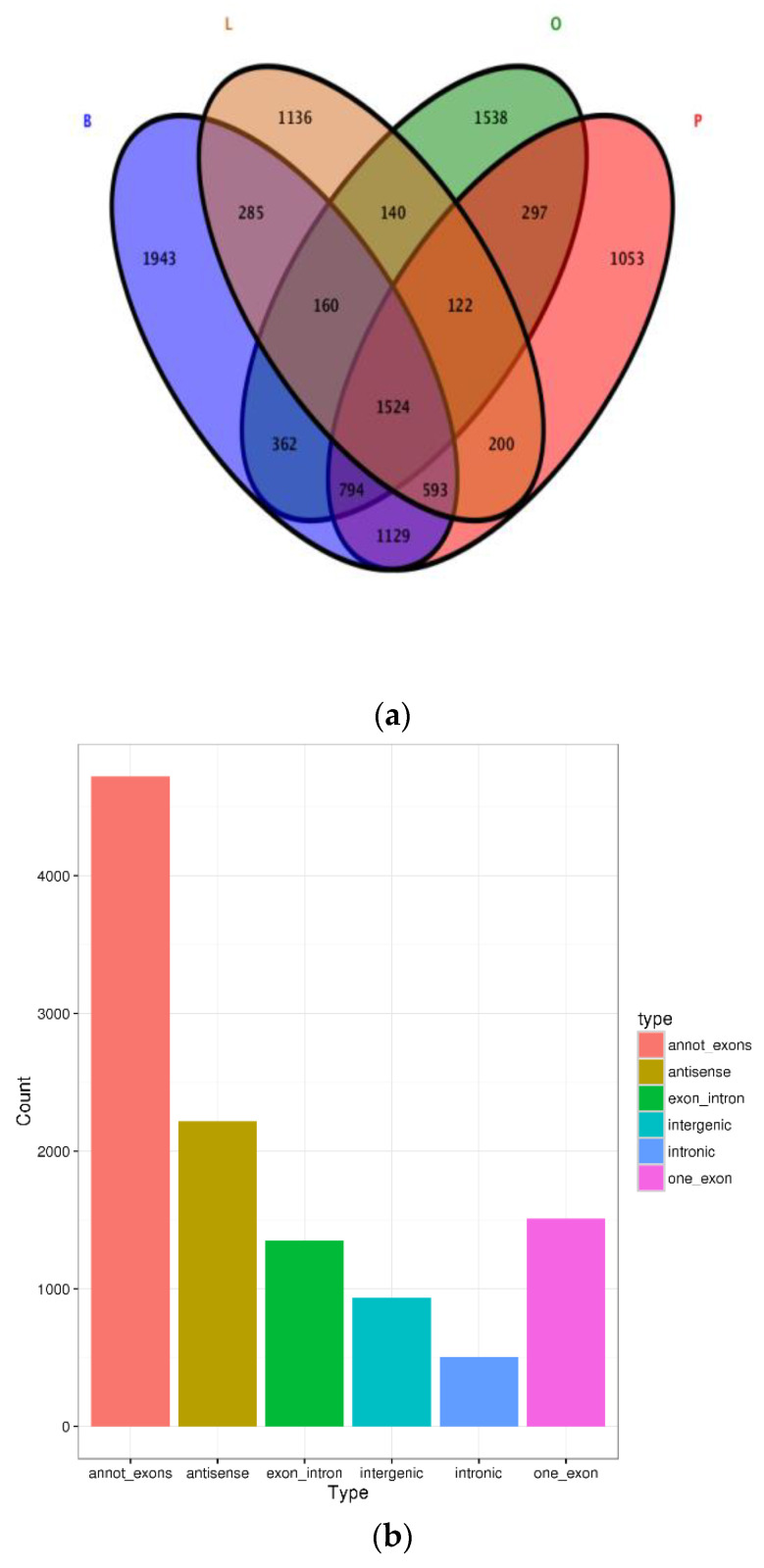

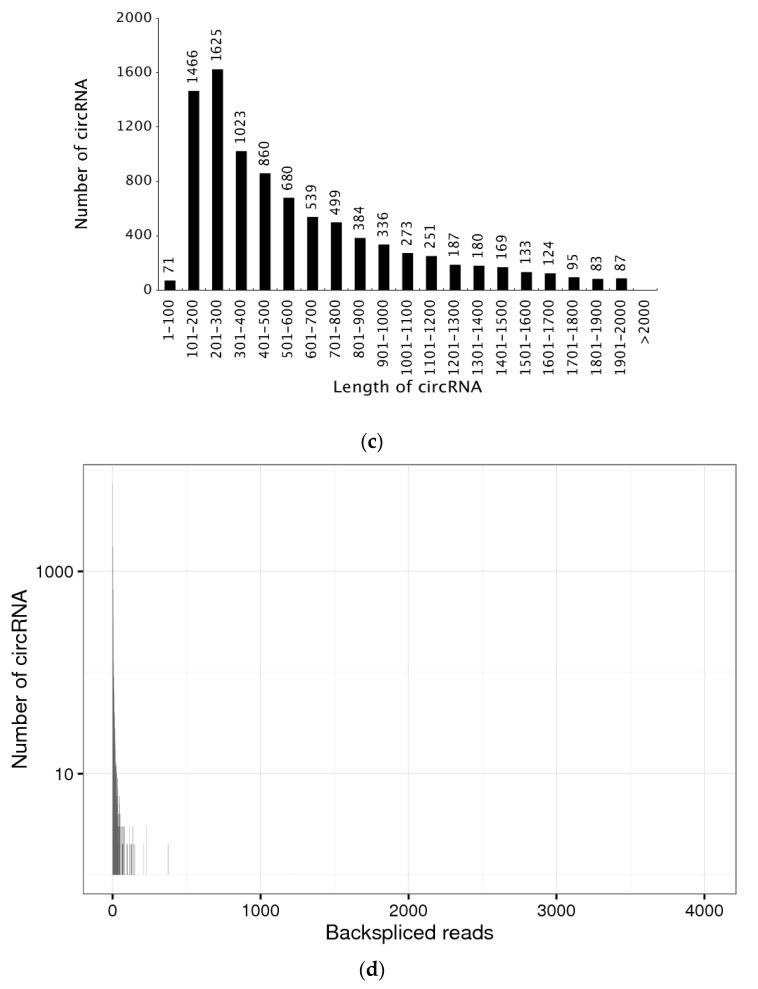

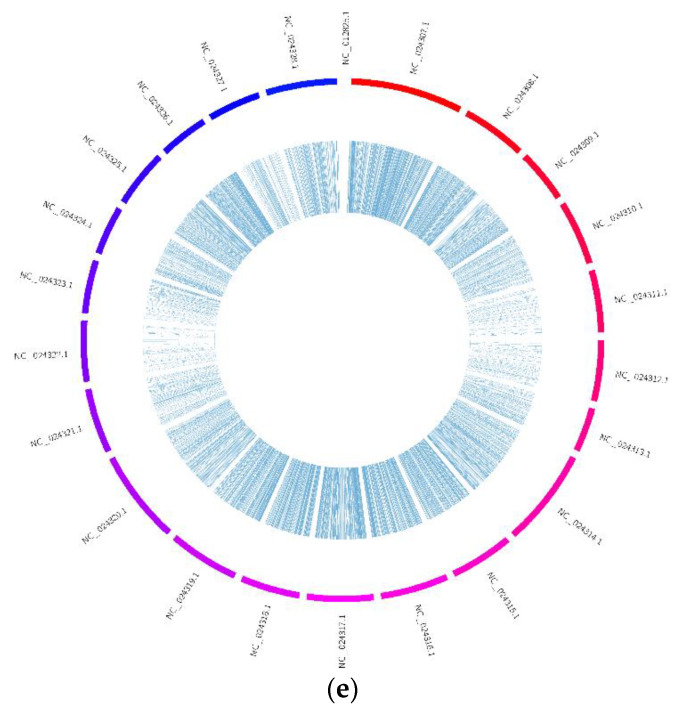
Statistics of circRNA. (**a**) Venn diagram of circRNAs distributed in different tissues, brain (B), pituitary (P), liver (L) and ovary (O). (**b**) The type of circRNAs. (**c**) The length of total circRNAs. (**d**) The number of back-spliced reads of circRNAs. (**e**) Distribution of circRNAs in different chromosomes.

We identified a considerable number of RNAs using high-throughput sequencing analysis of total RNA libraries of brain, pituitary, liver and ovary tissue across developmental stages IV, V and VI. In the libraries depleted of rRNA (rRNA−), among the total 11,276 distinct circRNA candidates that were generated in these tissues, we detected 6790 circRNAs in the brain, 4937 in the ovary, 5712 in the pituitary and 4160 in the liver, and determined the type of circRNA distribution. In addition, a total of 1524 circRNAs were identified in all four samples (Figure 1a). The number of putative circRNAs seemed to increase during the ovary maturation process (Figure S1a–c), suggesting a potential relationship between circRNAs and ovarian maturation in tongue sole. The total 6 types of circRNAs were found in the current study. We identified 4723 annot_exon circRNAs, 2216 antisense circRNAs, 1353 exon_intron circRNAs, 1509 one_exon circRNAs, 502 intronic circRNAs and 936 intergenic circRNAs, which occupied 42.02%, 19.72%, 12.04%, 13.43%, 4.47% and 8.32%, respectively (Figure 1b). The length distribution of most circRNAs was 101–500 nucleotides (nt), which was shorter than the mean transcript length of protein-coding genes (Figure 1c). The circRNA number of back-spliced reads in Figure 1d shows that most circRNAs back-spliced reads in 0–500. Notably, the expression levels of most circRNAs (n = 11,276) were not higher than 100 back-spliced reads (Figure 1d). We found the genomic loci from which circRNAs were derived to be widely distributed across chromosomes. However, the distribution of circRNAs detected in the current study was not uniform among different chromosomes (Figure 1e).

### 3.2. Identification of Differentially Expressed CircRNAs

**Figure 2:**
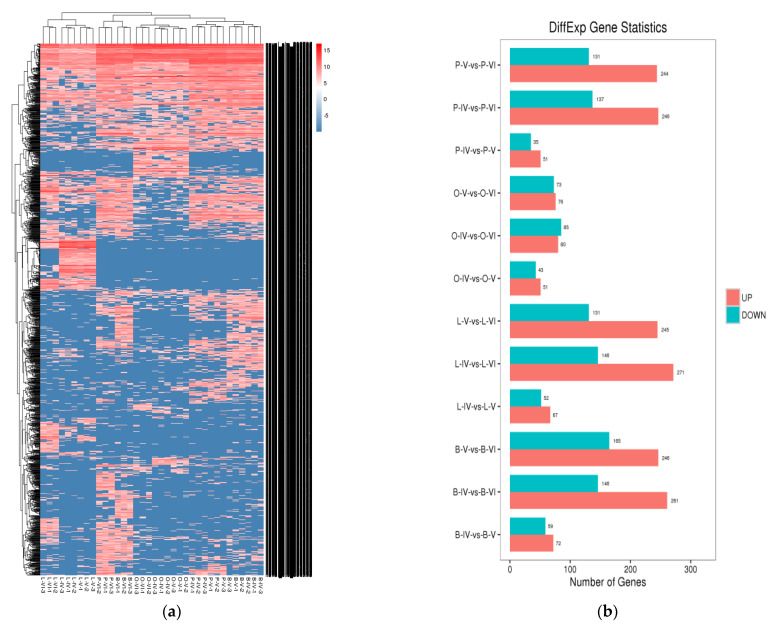
The total differentially expressed circRNAs. (**a**) Clustered heat map of differentially expressed circRNAs in the ovary (O), brain (B), pituitary (P) and liver (L) when comparing the IV-V, V-VI and IV-VI ovarian stages. (**b**) The statistical histogram of the number of differentially expressed circRNAs between different tissues and ovarian stages.

To further explore the potential functions of circRNAs, we prepared a clustered heat map of the differentially expressed circRNAs (Figure 2a). We divided the circRNAs in different comparison groups using circRNA expression analysis (Figure 2b). We found a specific number of circRNAs to be significantly (*p* < 0.05, |log_2_FC| > 1) differently expressed when comparing the libraries derived from the IV-V, IV-VI and V-VI comparison groups of the different tissues. While several circRNAs showed similar expression patterns, it became evident that a considerable number of circRNAs were differentially expressed not only in the comparison of different tissues but also among different developmental stages from the same samples. The results indicated that the circRNAs had organizational expression differences in the tongue sole and played an important role in the maturation of the ovary (Figure 2).

### 3.3. Analysis between CircRNAs and Their Parental Genes

**Figure 3:**
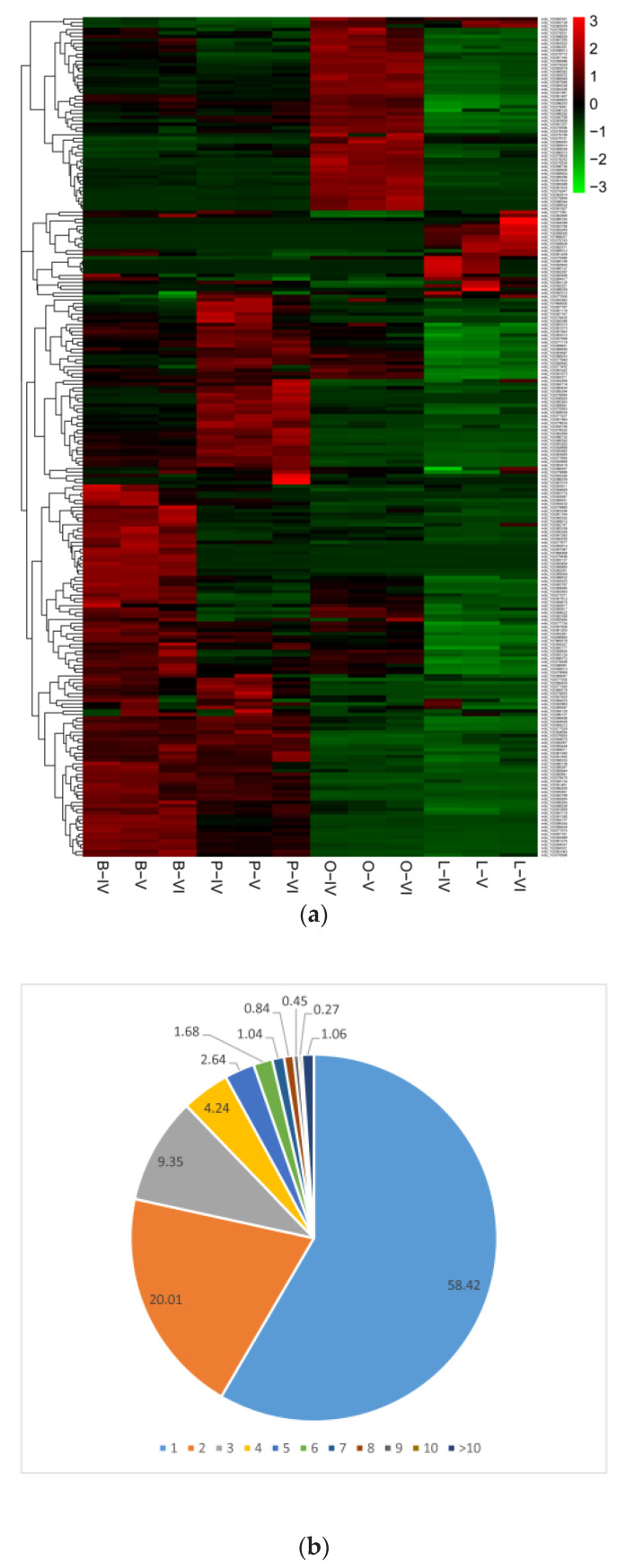

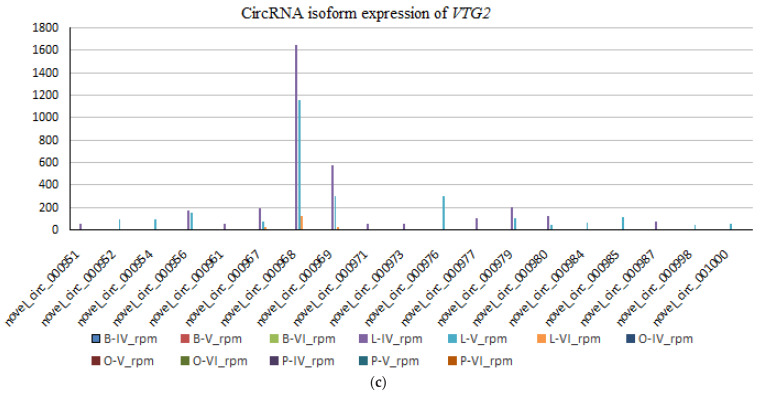
Characteristics of circRNAs in ovary (O), brain (B), pituitary (P) and liver (L) tissue of the ovarian stages IV, V and VI. (**a**) Clustered heat map showing abundances of the corresponding parental genes of the top 40 most differentially expressed circRNAs in the ovarian stages IV, V and VI in the ovary, brain, pituitary and liver. (**b**) Numbers of circRNAs produced by the same parental gene. (**c**) Example of *circ-VTG2*, which showed 19 alternative circRNA isoforms.

The corresponding parental genes of the top 40 most differentially expressed circRNAs were differentially expressed not only in the ovary, brain, pituitary and liver, but also among same samples in the ovarian stages IV-V, IV-VI and V-VI (Figure 3a). Notably, we discovered that some parent genes can generate multiple circRNA isoforms, which can exhibit expression profiles that are either positively or negatively correlated with those of the parent genes. Specifically, the 11,276 circRNAs detected in current study arose from only 4887 parental genes, and the different colours represent the number of circRNAs a parental gene can generate (Figure 3b). A striking example was the *VTG2* gene. *VTG2* has a regulatory effect on oocyte growth and maturation in fish, from which 19 different circRNA isoforms were detected. However, only one or two circRNA isoforms were expressed at higher levels in liver tissue, while the majority of isoforms showed low expression or no expression (Figure 3c).

### 3.4. Delineation of GO and KEGG Pathway Analysis

**Figure 4:**
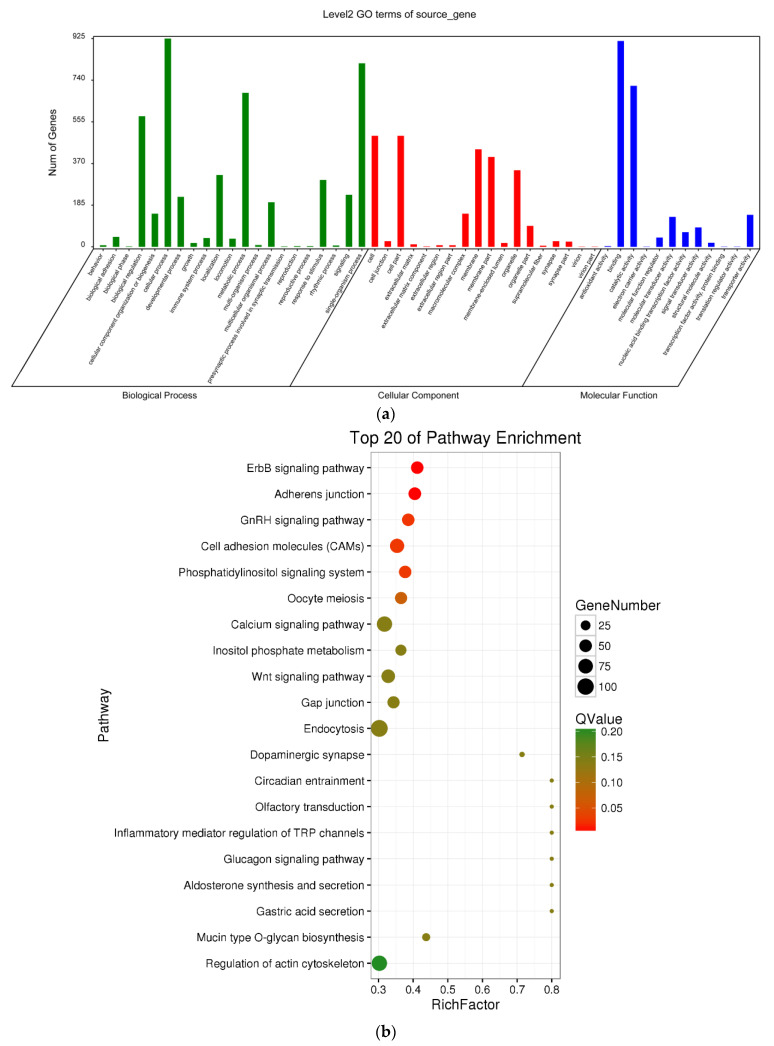
GO and KEGG pathways analyses. GO (**a**) and the top 20 KEGG (**b**) enrichment analyses of parental genes producing differentially expressed circRNAs.

As a first step to gain insight into whether circRNAs might regulate the transcription of their parent genes, in this work, we performed GO enrichment analysis of the genes that produced differentially expressed circRNAs. The most significant functional annotations are shown in Figure 4a. We found significant enrichment of 52 functional groups, which were classified into three GO categories: biological process, cellular component and molecular function (*p* < 0.05). Meanwhile, we employed KEGG pathway enrichment analysis to further understand the biological functions and molecular interactions of genes hosting significantly differentially expressed circRNAs, assuming that the identified pathways may participate in ovarian development and maturation. We found 170 pathways to be significantly enriched. The top 20 most significantly enriched KEGG pathways are shown in Figure 4b. We identified 6 different pathways (Q value < 0.05), namely, ErbB signalling pathway, adherens junction, GnRH signalling pathway, cell adhesion molecules (CAMs) and phosphatidylinositol signalling system. The GnRH signalling pathway may be related to ovarian maturation. In the differential analysis, the highest level of significance was found for ‘GnRH signaling pathway’ (ko04912), with 127 annotated genes, some of which were related to ovarian development and maturation. The results indicate that these enriched pathways may play an important role in the regulation of ovarian development and maturation in tongue sole.

### 3.5. CeRNA Network

We used the annotations of the functions of mRNAs to predict the potential functions of co-expressed circRNAs. The co-expression network revealed that one circRNA or mRNA correlated with one to dozens of differentially expressed circRNAs. In our study, we detected 11,276 circRNAs that arose from only 4887 parental genes and targeted 3186 miRNAs. There were 1,841,040 pairs of targeting relationships (Table 1).

**Figure 5:**
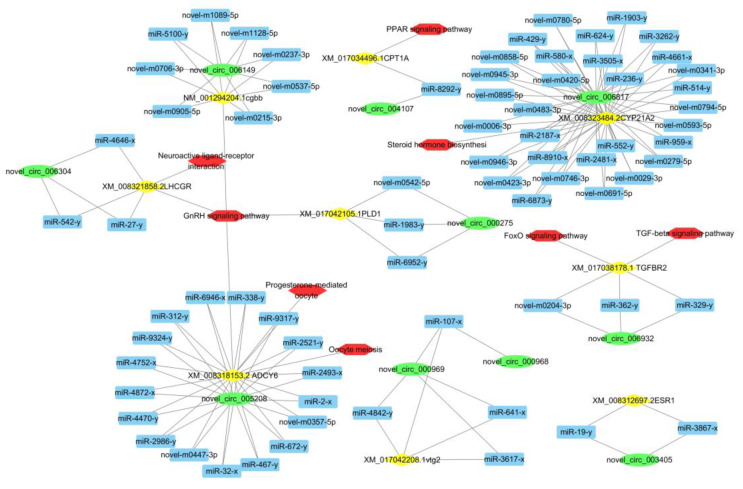
Construction of the circRNA-miRNA-mRNA network using online bioinformatics programmes (circBase, miRTarBase). The green circle indicates circRNA, the blue rectangle indicates miRNA, the yellow diamond indicates the targeted gene and the red hexagon indicates the gene pathway.

To construct a ceRNA network, based on network interaction, GO function and KEGG pathways, we focused on the pathways related to ovarian development and maturation: GnRH signalling pathway, oocyte meiosis, progesterone-mediated oocyte maturation, steroid hormone biosynthesis, TGF-beta signalling pathway and PPAR signalling pathway. We predicted their binding miRNAs using our high-throughput deep sequencing data. In view of shared binding sites in mRNAs and corresponding circRNAs, we constructed a circRNA-miRNA-mRNA co-expression network with 10 circRNAs, 9 mRNAs and 75 miRNAs, further confirming that circRNA-miRNA-mRNA interactions are not one-to-one targeting relationships (Figure 5). We found circRNAs that are associated with ovarian development and maturation, such as *circ-VTG2*, *circ-LHCGR*, *circ-ESR1* and *circ-LHβ*, suggesting that RNA interactions between circRNAs and mRNAs may play important roles in ovarian development and maturation.

### 3.6. Identification of CircRNAs

**Figure 6:**
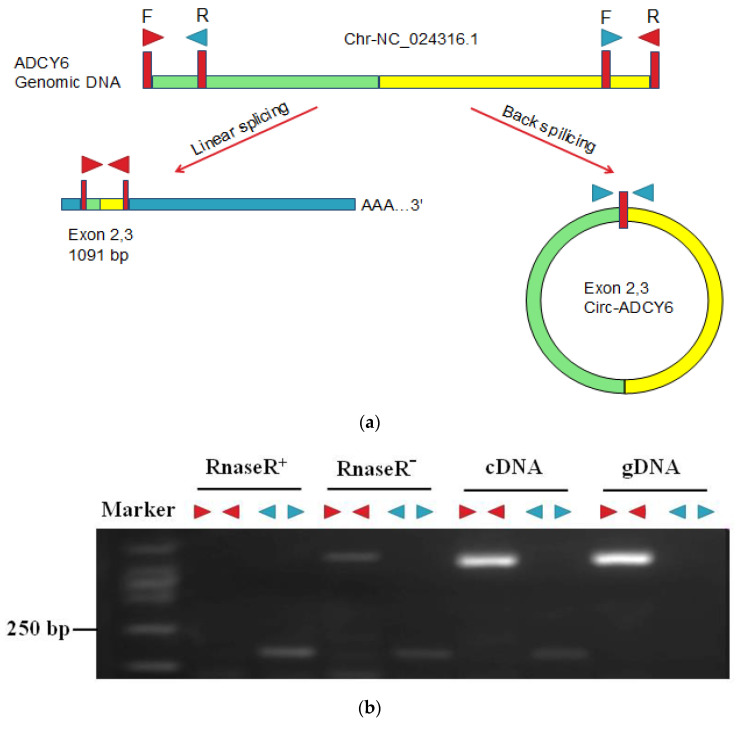

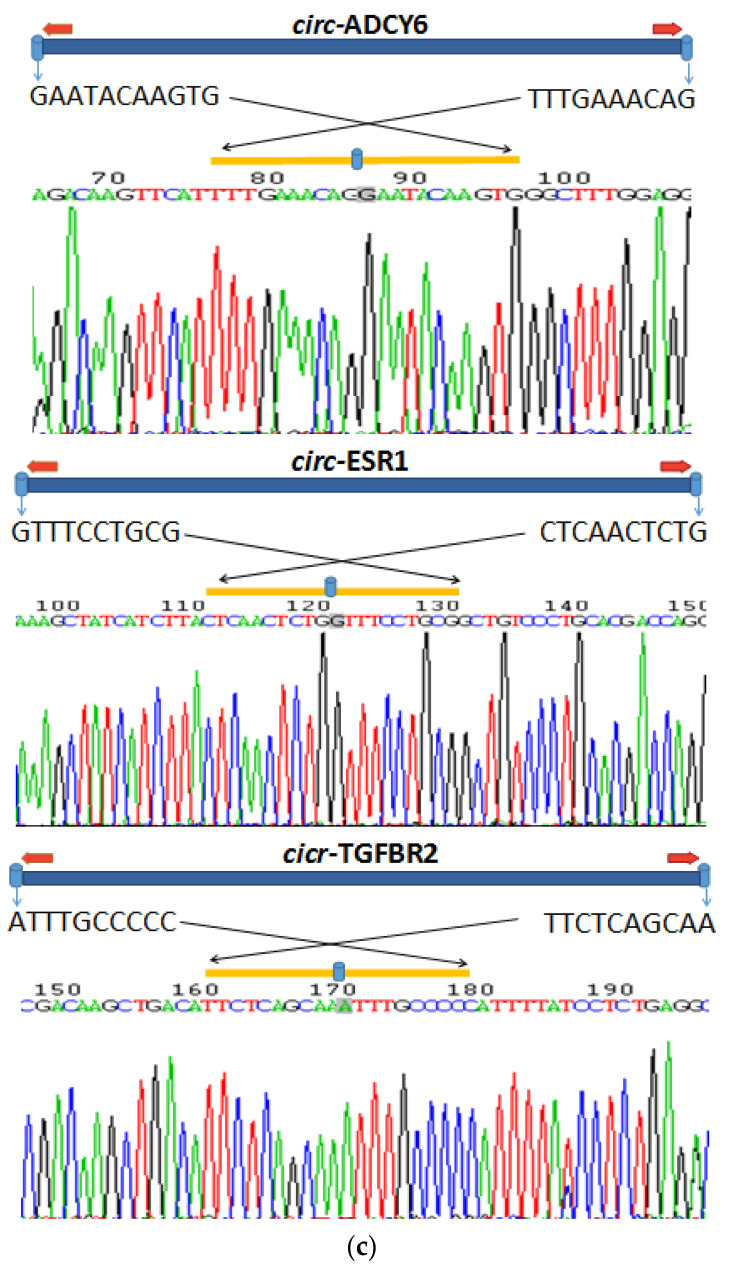
RNase R resistance assay. (**a**) Illustration of the annotated genomic region of *circ-ADCY6*. The putative different RNA splicing forms and convergent (red►◄) and divergent (blue◄►) primers were designed to amplify the linear and back-splicing products. (**b**) Total RNA from the brain with or without RNase R treatment was subjected to RT-PCR. Divergent and convergent primers were designed to amplify the back-splicing or linear products. (**c**) Representative examples of RT-PCR products sequenced to confirm circRNA junction sequences.

To verify that the *circ-TGFBR2*, *circ-ESR1* and *circ-ADCY6* genes are endogenous circRNAs, we designed convergent and divergent primers that specifically amplified the canonical or back-spliced forms of *circ-TGFBR2*, *circ-ESR1* and *circ-ADCY6* (Figure 6). We randomly selected and amplified *circ-ADCY6* junction regions using divergent primers and found that the *circ-ADCY6* back-spliced junction located between the 2 and 3 exons of the *ADCY6* genomic DNA (Figure 6a). Both R^+^ and R^−^ were reverse transcribed to cDNA using random primers. Convergent primers (red►◄) and divergent primers (blue◄►) were used to validate circRNAs and linear RNAs. PCR after reverse transcription with divergent primers detected *circ-ADCY6*, which was resistant to digestion by RNase R (Figure 6b). By comparison, convergent primers amplified the PCR products from linear *ADCY6* mRNA, which disappeared after RNase R digestion (Figure 6b). Sanger sequencing following RT-PCR conducted using the indicated divergent primers validated the “head-to-tail” splicing of *circ-ADCY6*, *circ-ESR1* and *circ-TGFBR2*. The Sanger sequencing of divergent primer-amplified products is shown in Figure 6c. The blue symbol on the yellow line indicates canonical back-splice sites.

### 3.7. Validation of Differentially Expressed CircRNAs by qRT-PCR

We verified the RNA-seq data using confirmed stage-specific differences in the abundance of certain circRNAs when comparing ovary, brain, pituitary and liver tissue samples from stages IV-V, V-VI and IV-VI using spanning junction primers to determine the relative expression levels of *circ-ADCY6*, *circ-TGFBR2*, *circ-CYP21A2*, *circ-CPT1A*, *circ-ESR1* and *circ-VTG2*(*novel_circ_000968*). The levels of six circRNAs in brain, pituitary, liver and ovary tissue samples from stages IV-V, V-VI and IV-VI were quantified by using qRT-PCR. The qRT-PCR validation test showed that the trend of expression of these circRNAs was basically in agreement with the results of sequencing (Figure S2), indicating that our deep-sequencing data are reliable. Moreover, these results showed a strong correlation between the data of RNA sequencing and qRT-PCR (r = 0.57, *p* < 0.05, Figure S3). Thus, overall, it suggested that circRNA-seq data provided reliable information about the relative abundance of circRNAs.

## 4. Discussion

Most studies examining the molecular mechanisms in humans and some animal and plant fungi have investigated the roles of protein-coding genes [24,25,26]. Accordingly, studies using RNA-seq analysis usually focus on mRNAs. With advances in high-throughput deep sequencing methods, RNA-seq assay and big data analysis, the abundance and diversity of circRNAs have been identified [27]. CircRNAs are novel RNA molecules with different biological functions and pathological implications [24]. Xu et al., (2017) used simulated traditional RNA-seq reads to evaluate the performance of CIRI and CIRCexplorer for circRNA identification and then confirmed the presence of 975 circRNAs in large yellow croaker and improved the understanding of the roles of circRNAs in teleosts [10]. However, to date, the potential functions of circRNAs in tongue sole ovary development and maturation and even in aquaculture have remained elusive. In this study, we investigated circRNAs to shed new light on tongue sole ovary development and maturation. Here, we provide for the first time an overview of the types and relative abundances of circRNAs that can be found in three reproductive stages of tongue sole’s ovary, brain, pituitary and liver tissue. Shen et al., (2017) identified 3868 circRNAs in zebrafish by using three algorithms (find_circ, CIRI, segemehl) and found that some circRNAs may function as miRNA sponges. Furthermore, the mechanism of zebrafish exonic circRNA biogenesis might be different from that in mammals [1]. Using high-throughput RNA-seq method, we discovered and annotated a large number of circRNAs in tongue sole and found that these circRNAs may participate in ovary development and maturation because their parental genes are closely related to these processes. We found that numerous circRNAs exist in the brain-pituitary-ovary and brain-pituitary-liver axes in tongue sole. A total of 11276 circRNAs were generated, of which 6790 circRNAs were detected in the brain, 5712 in the pituitary, 4937 in the ovary and 4160 in the liver. Most of the putative circRNAs we identified in tongue sole were expressed at low levels, which is consistent with recent studies [3,24] and suggests little potential function of most circRNAs [28,29,30,31]. In the current study, most circRNAs were derived from exons of genes, which was similar to those in human organs [32,33]. However, tongue sole exonic circRNA biogenesis is to some extent different from that reported by Shen et al., (2017) [1].

CircRNAs have been shown to function as miRNA sponges. Li et al., (2018) found that circFGFR4 promotes the differentiation of myoblasts by binding miR-107 to relieve its inhibition of Wnt3a [34]. Wang et al., (2019) found that the circRNA circP4HB enhances epithelial-mesenchymal transition and metastatic disease in humans by sponging miR-133a-5p, leading to upregulation of vimentin expression. Only a few of these circRNAs have been reported to have functions [35]. In our study, we also found that circRNAs have the spongy effect of miRNAs, consistent with the results of relevant studies. We found that the mRNA-miRNA-circRNA interaction is not a one-to-one targeting relationship. Our results indicated that miRNAs and genes interacted with each other through a complex regulatory network. For example, miR-19-y was sponged by *circ-ESR1*, which sponged another miRNA (miR-3867-x) in the network. In the current study, 4887 parental genes were first identified to produce 11276 circRNAs, targeting 3186 miRNAs, resulting in 1841040 pairs. One parental gene can produce one or more circRNA, which implies the vital function of circRNA temporal regulation. This is consistent with the research results of goats (*Capra hircus*) [17]. Thus, the most possible relationship of the expression between the circRNAs and their parent genes in fishes seems complexity and merits further investigation.

In contrast with linear RNAs, circRNAs show higher tolerance to exonucleases because of their covalently linked circular structure. The stability, conservation, abundance and tissue specificity of circRNAs make them ideal candidates for molecular markers in some pathologic processes [36,37,38]. Exon-derived circRNAs are predominantly localized in the cytoplasm and likely function via a variety of mechanisms [39,40]. Some circRNAs have been reported to be potential diagnostic biomarkers in ovarian cancer. Among them, 6 circRNAs (circBNC2, circEXOC6B, circFAM13B, circN4BP2L2, circRHOBTB3 and circCELSR1) have been associated with various clinicopathological features of epithelial ovarian cancer (EOC). More importantly, circEXOC6B and circN4BP2L2 may act as novel diagnostic biomarkers in patients with EOC [41]. Another study investigated circRNA expression profiles in EOC and showed that a total of 4388 circRNAs were differentially expressed in EOC tissues compared with normal ovarian tissues, of which 2556 were upregulated and 1832 were downregulated. The authors also found that circEXOC6B and circN4BP2L2 may act as novel prognostic biomarkers in patients with EOC [42]. Our results verified that circRNAs such as *circ-ADCY6*, *circ-ESR1* and *circ-TGFBR2* were resistant to RNase R. Considering their resistance to RNase R digestion treatment, circRNAs may be useful potential biomarkers for screening and predicting the prognosis of human diseases [43,44,45,46]. Further studies are needed to analyze circRNA functions and molecular mechanisms in the ovary development and maturation of tongue sole and to discuss whether circRNAs can be used as emerging genetic markers in the ovary development and maturation in fish.

The circRNAs in tongue sole showed some similar but also distinct features from circRNAs from other species. To date, many circRNAs have been found in various human, animal and plant tissues or at different developmental stages. Researchers have identified 159493 unique human circRNA candidates thus far [32]. It seems that there is a potential relationship between circRNAs and tomato fruit ripening since the total number of putative circRNAs was increased [28]. A few circRNAs we validated were upregulated in our study, which is similar to the expression of their parent genes during the ripening process of tomato. A study of *Ganoderma lucidum* suggested additional layers of interactions among circRNA isoforms and their parent genes in terms of gene expression regulation [47]. A previous study found that circRNAs were abundant and spatiotemporally specific during human ovarian ageing [32]. We identified a number of circRNAs in different reproduction-related tissues during tongue sole ovarian development and maturation and found that circRNAs also have spatiotemporal specificity, consistent with human studies. In a study of soybean (*Glycine max* (Linn.) Merr.), the majority of circRNAs were specifically expressed in different tissues, and the circRNA isoforms generated by alternative circularization were also tissue preferentially expressed [26]. These results are consistent with our findings.

In our study, the genes we chose to focus on were related to ovarian development and maturation from different expression pathways. We found that *circ-ADCY6*, *circ-TGFBR2*, *circ-ESR1*, *circ-LHβ*, *circ-CYP21A2*, *circ-CPT1A*, *circ-PLD1*, *circ-VTG2* (*novel_circ_000968*) and *circ-LHCGR* may play an important regulatory role in ovarian development and maturation. Relevant studies have shown that most of the Vtg of bony fishes is produced in the liver [48], and *circ-VTG2* (*novel_circ_000968*) in our study was also differentially expressed in the liver. Couse et al., (1999) [49] and Lubahn et al., (1993) [50] have shown that *ESR1* is closely related to the development and functional maintenance of female mouse ovaries. Li et al., (2013) found that tongue sole *ER* is involved in the development and maturation of the testis [51]. We speculated that *circ-ESR1*, which we found in the present study, may have a regulatory effect on *ESR1* in tongue sole and further inferred that *circ-ESR1* has a regulatory function in ovarian development in tongue sole. Liu et al., (2013) found that the expression of *LHCGR* in cultured zebrafish follicle cells and its biphasic response to estrogens [52]. Wohlres et al., (2019) found that downregulation of total *LHCGR* and the associated with upregulation of their inactive isoforms may compromise follicle development and thus contribute to the low efficiency of superovulation in heifers (*Gir cattle*) with a poor responder phenotype [53]. Shi et al., (2015) suggested that *FSH* and *LH* may simultaneously regulate ovarian development and maturation through the brain-pituitary-ovary axis endocrine system in tongue sole [20]. We thus hypothesized that *circ-LHCGR* and *circ-FSH* might also share similar mechanisms with their parent genes in the ovarian development and maturation of tongue sole.

Tao et al., (2018) found that chi-circ_0008219 sponges 3 ovarian follicle-related miRNAs, and the findings demonstrate that circRNAs have potential effects in the ovarian follicles of goats and may represent a promising new research field in ovarian follicular development [17]. Guo et al., (2019) demonstrated that circINHA promoted granulosa cell (GC) proliferation and inhibited GC apoptosis via connective tissue growth factor (CTGF) by acting as a ceRNA that directly binds to miR-10a-5p [54]. This study presents evidence for the circINHA/miR-10a-5p/CTGF regulatory pathway in follicular GC apoptosis and provides novel insights into the role of circRNAs in the modulation of ovarian physiological functions. Increasing numbers of research have shown that circRNAs play vital roles in ovarian development and maturation. Based on parent gene GO function and KEGG pathway analysis, we found that some circRNAs were closely associated with biological processes involved in key signalling pathways, such as the GnRH signalling pathway, oocyte meiosis, progesterone-mediated oocyte maturation, steroid hormone biosynthesis, TGF-beta signalling pathway and PPAR signalling pathway, which may play pivotal roles in ovarian development and maturation. Furthermore, several hundred circRNAs were significantly changed during ovary development and maturation in tongue sole.

In the ovaries, the steroid biosynthesis pathway produces various steroid hormones related to reproduction and plays an indispensable role in the reproductive process. In goats, GO and KEGG pathway analyses revealed that many target genes were enriched in the p53 signalling pathway and the ovarian steroidogenesis pathway and exhibited a strong relationship with the biological processes of goat ovarian follicle development and reproductive characteristics [17]. In our study, we identified several differentially expressed genes in ovarian development and maturation, such as *CYP21A2*, *VTG2* and *ESR1,* which come from the steroid biosynthesis pathway. According to GO and KEGG pathway analyses in the ovary of honey bees (*Apis mellifera*), the parent genes of differentially expressed circRNAs regulate various biological and reproduction-related processes, including calcium signalling pathways and insulin secretion. Other biological processes in ovary activation and oviposition were also enriched, such as the neuroactive ligand-receptor interaction, Hippo signalling pathway, Wnt signalling pathway and MAPK pathways [30]. The KEGG bioinformatics analysis results by Shen et al., (2019) indicated that circRalGPS2s was significantly involved in the pathways tight junctions, GnRH signalling, progesterone-mediated oocyte maturation and FoxO signalling, which further explains the role of these pathways as key pathways in follicle development [55]. Previous results suggested that the differential expression of circRNAs involved in these pathways may be related to ovarian development and maturation. In current study, we identified *ADCY6* in the progesterone-mediated oocyte maturation pathway, and *ADCY6* was differentially expressed in the GnRH signalling pathway and oocyte meiosis, two key pathways involved in ovarian development and maturation. We found that the *TGFBR2* hub gene was actively associated with the TGF-beta and FoxO signalling pathways. The FoxO signalling pathway has been reported to be a key mediator of the proliferation of GCs [56]. In the current study, *PLD1* and *LH-β* were differentially expressed in the GnRH signalling pathway. *CPT1A* was differentially expressed in the PPAR signalling pathway. *LHCGR* was differentially expressed in neuroactive ligand-receptor interaction and calcium signalling pathway. Consistent with previous studies, our study demonstrated the presence of differentially expressed circRNAs in the signalling pathways related to ovarian development and maturation in tongue sole, suggesting a possible relationship between circRNAs and these pathways. Our KEGG analysis results showed that the GnRH signalling pathway, oocyte meiosis and calcium signalling pathway were differentially expressed pathways. We speculated that circRNAs have significant biological functions in ovarian development and maturation in tongue sole.

## 5. Conclusions

For the first time, we explored the abundance and characteristics of circRNAs from different developing stage and in the ovary, brain, pituitary and liver tissues of tongue sole using high-throughput deep sequencing technology. In addition, we found that most of the putative circRNAs were expressed at low levels and derived from gene exons. CircRNAs and circRNA isoforms exhibited tissue-specific and stage-specific expression patterns, and we selected *circ-ADCY6*, *circ-TGFBR2*, *circ-CPT1A*, *circ-ESR1*, *circ-VTG2* (novel_circ_000968) and *circ-CYP21A2* from the KEGG pathway of ovarian development and maturation. qRT-PCR largely confirmed the differential expression patterns. The co-expression network revealed that one mRNA correlates with one to dozens of differentially expressed circRNAs, while the majority of isoforms showed low expression or no expression. CircRNA was ring forming and had RNase R tolerance.

Our study is the first to delineate the expression profile of circRNAs in the brain-pituitary-liver and brain-pituitary-ovarian axes during the process of ovarian development and maturation in tongue sole. In the future, we will focus on the functional analyses of identified circRNAs in ovarian development and maturation in tongue sole.

## Figures and Tables

**Table 1:** CircRNA new predictive targeting relationship.

miRNA	CircRNA	mRNA	Number of Targets
3186	11,276	4887	1,841,040

## Data Availability

The data presented in this study are available on request from the corresponding author.

## Supplementary Materials

The following are available online at www.mdpi.com/article/10.3390/biology10090830/s1. Figure S1: Distribution of circRNAs in tongue sole at different ovarian stages. Figure S2: Comparison between qRT-PCR validation results and RNA-Seq data. Figure S3: Coefficient analysis of fold-change data from qRT-PCR and RNA-seq. Table S1: Nucleotide sequences of primers used for PCR amplification of circRNAs. Table S2: qRT-PCR primers of circRNAs. Table S3: Summary of circRNA sequencing data.
