# Supplementary material for: Comprehensive CircRNA Profiling and Selection of Key CircRNAs Reveal the Potential Regulatory Roles of CircRNAs throughout Ovarian Development and Maturation in Cynoglossus semilaevis
Source: Biology (Basel). 2021 Aug 26;10(9):830. doi: 10.3390/biology10090830 (PMC8468179; doi:10.3390/biology10090830)
Supplement: Supplementary file 1 [file biology-10-00830-s001.zip › biology-1272767-supplementary.pdf]

biology

MDPI

a

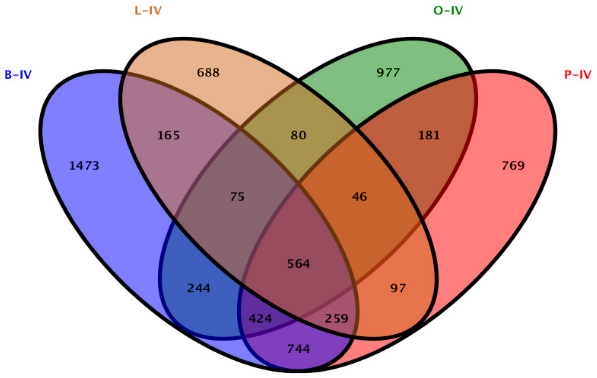

b

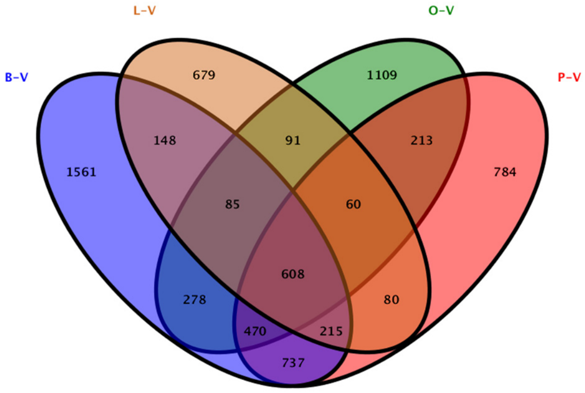

c

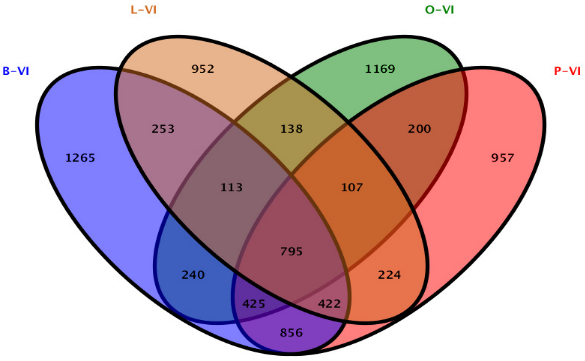

Figure S1. Distribution of circRNAs in tongue sole at different ovarian stages. B: brain, P: pituitary, L: liver, O: ovary. (**a**) Distribution of circRNAs in tongue sole at ovarian stage IV. (**b**) Distribution of circRNAs in tongue sole at ovarian stage V. (**c**) Distribution of circRNAs in tongue sole at ovarian stage VI.

a

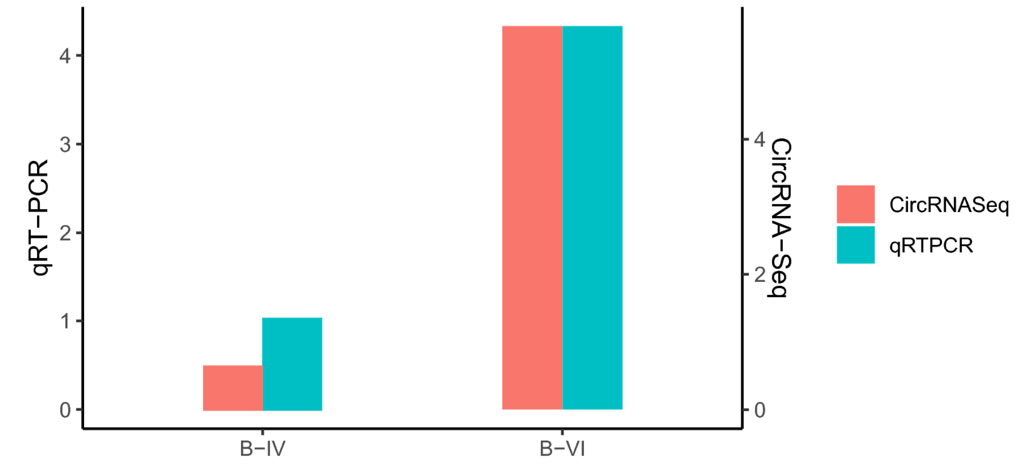
qRT−PCR
4
3
2
1
0
B−IV
B−VI
CircRNA−Seq
4
2
0
CircRNASeq
qRTPCR

b

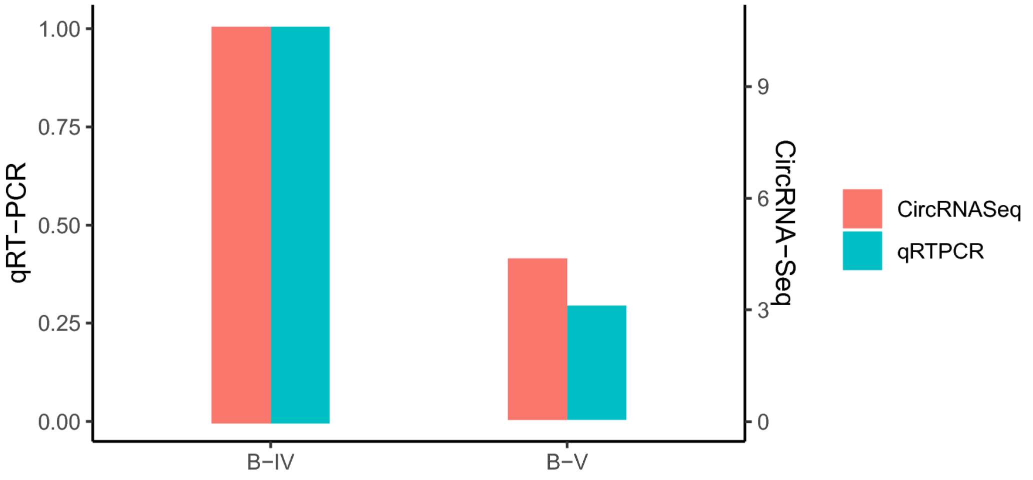
qRT−PCR
1.00
0.75
0.50
0.25
0.00
B−IV
B−V
CircRNA−Seq
9
6
3
0
CircRNASeq
qRTPCR

c

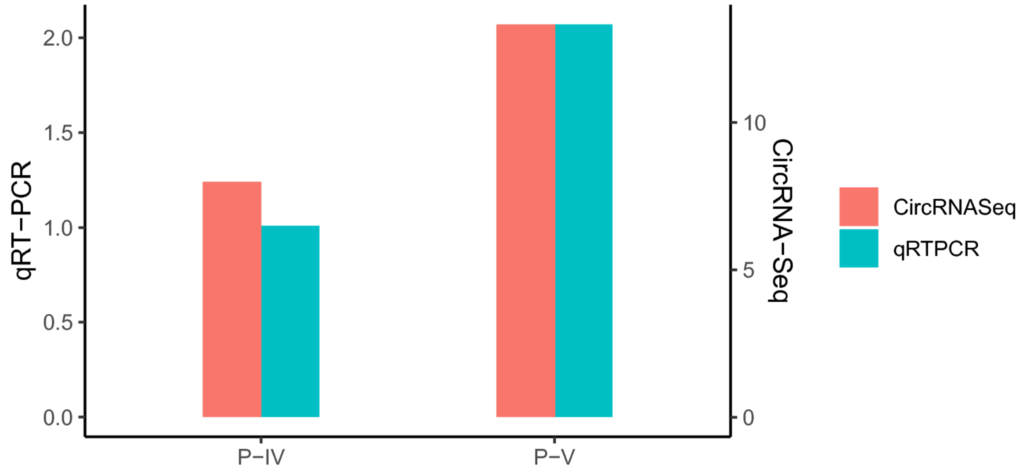
qRT−PCR
2.0
1.5
1.0
0.5
0.0
P−IV
P−V
CircRNA−Seq
10
5
0
CircRNASeq
qRTPCR

d

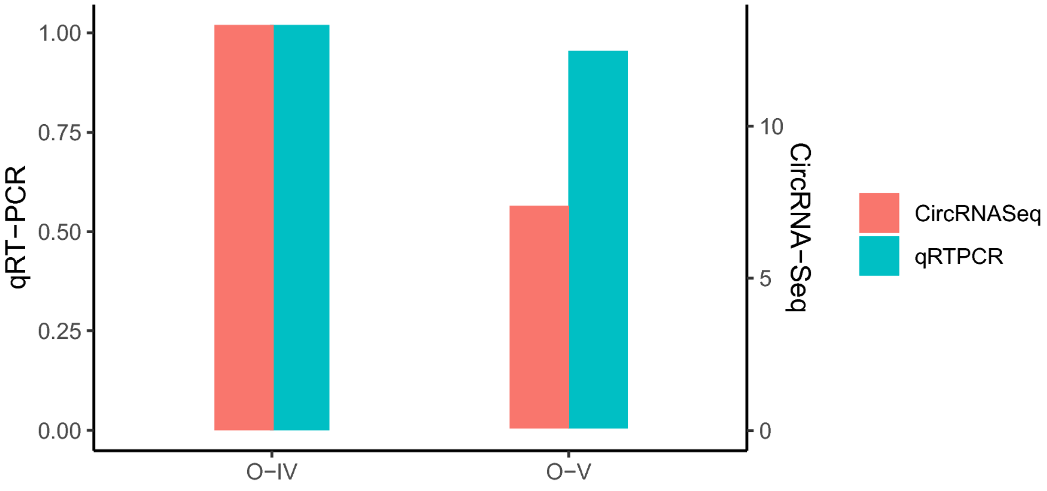
1.00
0.75
0.50
0.25
0.00
qRT−PCR
10
5
0
CircRNA−Seq
O−IV
O−V
CircRNASeq
qRTPCR

e

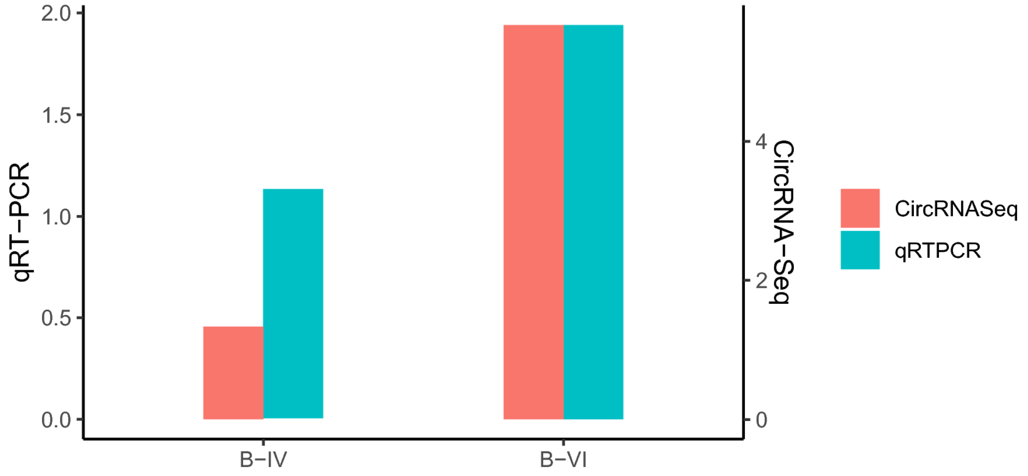
2.0
1.5
1.0
0.5
0.0
qRT−PCR
4
2
0
CircRNA−Seq
B−IV
B−VI
CircRNASeq
qRTPCR

f

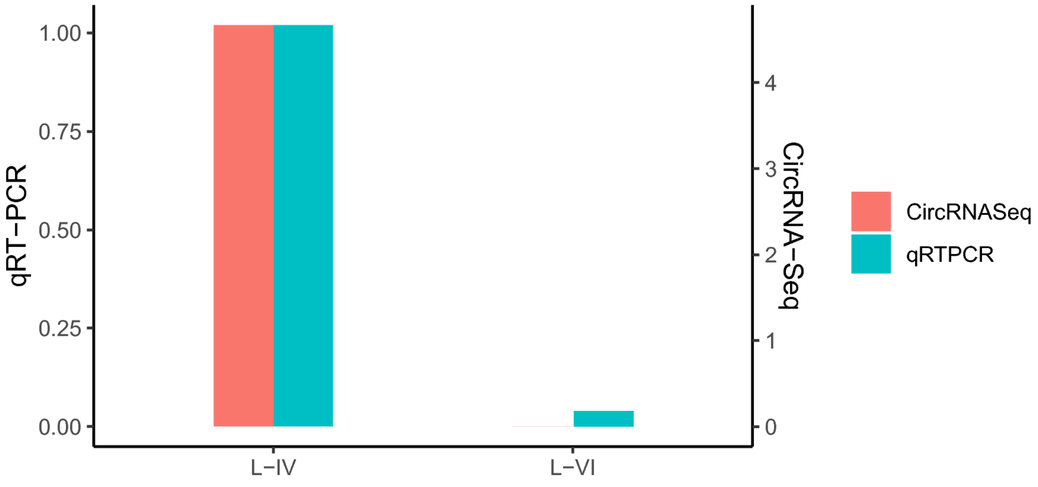
1.00
0.75
0.50
0.25
0.00
qRT−PCR
4
3
2
1
0
CircRNA−Seq
L−IV
L−VI
CircRNASeq
qRTPCR

g

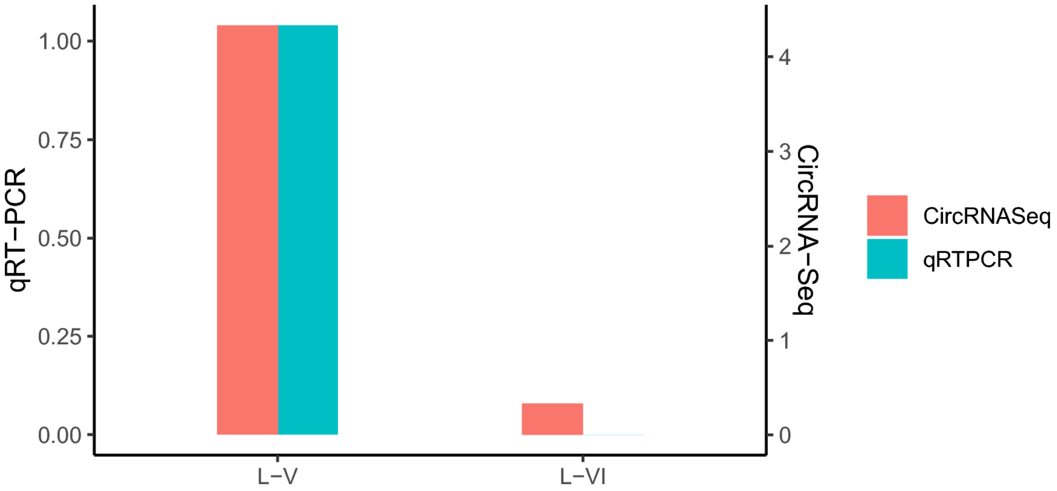

Figure S2. Comparison between qRT-PCR validation results and RNA-Seq data (**a**: *circ-ADCY6*, **b**: *circ-TGFBR2*, **c**: *circ-CYP21A2*, **d**: *circ-CYP21A2*, **e**: *circ-CPT1A*, **f**: *circ-ESR1*, **g**: *circ-VTG2*). B: brain, P: pituitary, L: liver, O: ovary.

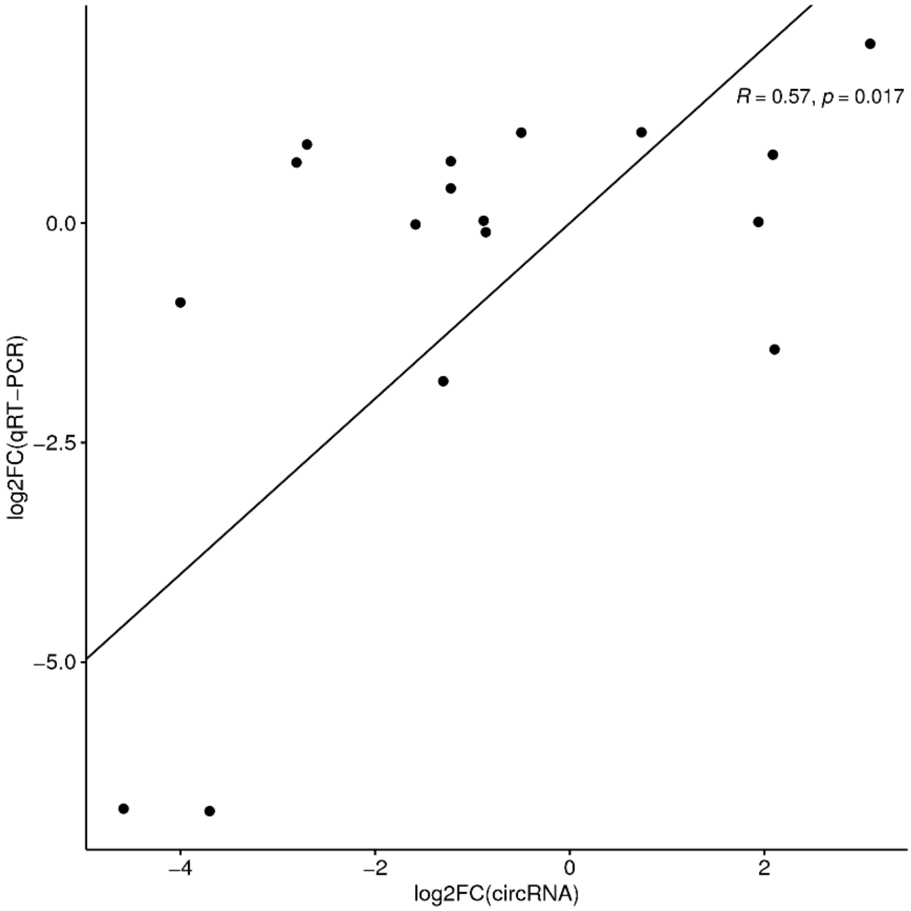

Figure S3. Coefficient analysis of fold-change data from qRT-PCR and RNA-seq. Six cirRNAs were selected for qRT-PCR. Scatterplots were generated from the expression ratios obtained from RNA-seq (X-axis) and qRT-PCR (Y-axis).

Table S1. Nucleotide sequences of primers used for PCR amplification of circRNAs.

CircRNA ID	Convergent primers (5' - 3')	Divergent primers (5' - 3')
ADCY6	F: GAATACAAGTGGGCTTTGGAGGC<br>R: TGCCAGGCGGTGACGATGT	F: CACTGCTGCCGGTCAGAATG<br>R: GCCTCCAAAGCCCACTTGTATT
ESR1	F: CCTGGTTAGAGGTGCTGATGATTGG<br>R: TGCGGAAGCGGGAAGTGGTG	F: ATCTCATACTGGACAGGAATGAAGGCAACT<br>R: AACCTGGTCGTGCAGGGACAGC
TGFBR2	F: CGTAGAACTCGCCTCGTGC<br>R: GTGGCTTGGAAGGGTTATGG	F: CTGGAGAAAGAACGAGACCG<br>R: TGGAAAGCAGCGAGGTG
β-actin	F: GGTCTGTGATGCCCTTAGATGTC<br>R: AGTGGGGTTCAGCGGGTTAC	

Table S2. qRT-PCR primers of circRNAs.

CircRNA ID	Primer sequence (5' - 3')
Circ-CPT1A-F	AATGGGAAGAACGGCATCAAC
Circ-CPT1A-R	TGGAAACAGTCAGTGGCTAAAGTG
Circ-CYP21A2-F	CGCACACACACATCTTCACACTAA
Circ-CYP21A2-R	CAGACCCATGGACAGGTGTAG
Circ-ADCY6-F	TCAGAATGAGAGCAGCCGTC
Circ-ADCY6-R	GAGTAGGCGCTCCTGTTTCA
Circ-TGFBR2-F	CGAGCGCATCTCCACGTTAG
Circ-TGFBR2-R	CGATCTCGCTCCTTCTCAGG
Circ968-Vtg2-F	AGCTCATGGATAGGCTGACG
Circ968-Vtg2-R	AATCACGCTTGCCCTGCTAA
Circ-PLD1-F	TTTCTTGCTGCGGTATTGGC
Circ-PLD1-R	GGCCCATGGAGCACTGAATTA
β-actin-F	GCACGGTATTGTGACCAACTGG
β-actin-R	CAGGGGAGCCTCTGTGAGC

Table S3. Summary of circRNA sequencing data. B: brain, P: pituitary, L: liver, O: ovary. IV: ovarian stage IV. V: ovarian stage V. VI: ovarian stage VI.

Sample	Raw data (bp)	Clean Data (bp)	Q20 (%)	Q30 (%)	N (%)	GC (%)
B-IV-1	11687839200	11337553205	11109658829 (97.99%)	10628681372 (93.75%)	278679 (0.00%)	5588501053 (49.29%)
B-IV-2	11236317900	10902495646	10675964629 (97.92%)	10202547873 (93.58%)	268101 (0.00%)	5274077107 (48.37%)
B-IV-3	11638397700	11275184758	11036657006 (97.88%)	10541858631 (93.50%)	277417 (0.00%)	5494620501 (48.73%)
P-IV-1	11397208800	11022688918	10808912012 (98.06%)	10355005195 (93.94%)	270017 (0.00%)	5665761799 (51.40%)
P-IV-2	12329510100	11969867228	11728538841 (97.98%)	11224311049 (93.77%)	293260 (0.00%)	6140764477 (51.30%)
P-IV-3	11907719100	11539952887	11308559354 (97.99%)	10823272494 (93.79%)	283761 (0.00%)	5892539015 (51.06%)
O-IV-1	11885582700	11515382363	11281304136 (97.97%)	10793119170 (93.73%)	282761 (0.00%)	5971480899 (51.86%)
O-IV-2	11189614800	10825566692	10599988549 (97.92%)	10135878788 (93.63%)	265323 (0.00%)	5687014591 (52.53%)
O-IV-3	11430917700	11096828311	10882905774 (98.07%)	10431456999 (94.00%)	273359 (0.00%)	5703261966 (51.40%)
L-IV-1	11391219600	11103662320	10902759215 (98.19%)	10469149417 (94.29%)	274099 (0.00%)	5765561884 (51.92%)
L-IV-2	12230671200	11911593806	11665137836 (97.93%)	11134965260 (93.48%)	129459 (0.00%)	6345678562 (53.27%)
L-IV-3	12567467100	12271323131	12058578220 (98.27%)	11597988436 (94.51%)	302472 (0.00%)	6755260689 (55.05%)
B-V-1	14049978600	13653968437	13350284065 (97.78%)	12705001688 (93.05%)	149247 (0.00%)	6581841019 (48.20%)
B-V-2	13437271500	13064378709	12779086938 (97.82%)	12168811148 (93.14%)	141537 (0.00%)	6316558324 (48.35%)
B-V-3	14180755800	13776876754	13470286463 (97.77%)	12818547160 (93.04%)	148764 (0.00%)	6636189602 (48.17%)
P-V-1	11707022400	11327399444	11063823525 (97.67%)	10514173415 (92.82%)	88096 (0.00%)	5687787051 (50.21%)
P-V-2	11559978900	11212961657	10991295060 (98.02%)	10522506448 (93.84%)	276973 (0.00%)	5564522422 (49.63%)
P-V-3	11770452900	11378998281	11094979655 (97.50%)	10514485971 (92.40%)	136851 (0.00%)	5657951012 (49.72%)
O-V-1	12581521200	12211619999	11932884351 (97.72%)	11345312689 (92.91%)	143361 (0.00%)	6097317736 (49.93%)
O-V-2	13292656200	12904213930	12642801889 (97.97%)	12095121425 (93.73%)	316478 (0.00%)	6597144733 (51.12%)
O-V-3	12085141200	11799150178	11628204296 (98.55%)	11234260559 (95.21%)	44068 (0.00%)	6101259977 (51.71%)
L-V-1	12392845200	12167082991	12045626552 (99.00%)	11734985218 (96.45%)	108176 (0.00%)	6663959206 (54.77%)
L-V-2	14059226700	13688367914	13393830409 (97.85%)	12766500339 (93.27%)	148020 (0.00%)	7264764337

						(53.07%)
L-V-3	13846496400	13524127596	13261647208 (98.06%)	12680534663 (93.76%)	146561 (0.00%)	7128503699 (52.71%)
B-VI-1	11164143000	10845080636	10627923167 (98.00%)	10169206543 (93.77%)	267098 (0.00%)	5220039588 (48.13%)
B-VI-2	10992373500	10679564530	10462555894 (97.97%)	10003837123 (93.67%)	262295 (0.00%)	5052499278 (47.31%)
B-VI-3	11802252900	11426129969	11162414319 (97.69%)	10607414013 (92.83%)	135280 (0.00%)	5505954447 (48.19%)
P-VI-1	11680254600	11319933392	11055864953 (97.67%)	10507065745 (92.82%)	123369 (0.00%)	5566275021 (49.17%)
P-VI-2	13062954000	12683640819	12405877580 (97.81%)	11835124401 (93.31%)	310898 (0.00%)	6308874840 (49.74%)
P-VI-3	12595436400	12243753475	11994573762 (97.96%)	11470926925 (93.69%)	301195 (0.00%)	6144015448 (50.18%)
O-VI-1	12142121700	11658952372	11284277118 (96.79%)	10598088261 (90.90%)	126387 (0.00%)	5680944490 (48.73%)
O-VI-2	12281954400	11907393064	11632547204 (97.69%)	11053657571 (92.83%)	149681 (0.00%)	5902073601 (49.57%)
O-VI-3	12730120800	12343537306	12070704664 (97.79%)	11492288166 (93.10%)	134352 (0.00%)	6102729718 (49.44%)
L-VI-1	13445492700	13069939112	12785172279 (97.82%)	12182140951 (93.21%)	140462 (0.00%)	6987059285 (53.46%)
L-VI-2	13826369100	13448497075	13148072489 (97.77%)	12514984499 (93.06%)	145382 (0.00%)	7032050682 (52.29%)
L-VI-3	13581389700	13257672890	12983766650 (97.93%)	12386943624 (93.43%)	143709 (0.00%)	6797473707 (51.27%)
